# Association between Reactogenicity and Immunogenicity after Vaccination with BNT162b2

**DOI:** 10.3390/vaccines9101089

**Published:** 2021-09-27

**Authors:** Stilla Bauernfeind, Bernd Salzberger, Florian Hitzenbichler, Karolina Scigala, Sebastian Einhauser, Ralf Wagner, André Gessner, Josef Koestler, David Peterhoff

**Affiliations:** 1 Department of Infection Prevention and Infectious Diseases, University Medical Center Regensburg, 93053 Regensburg, Germany; bernd.salzberger@ukr.de (B.S.); florian.hitzenbichler@ukr.de (F.H.); karolina.scigala@stud.uni-regensburg.de (K.S.); 2 Institute for Medical Microbiology and Hygiene, University of Regensburg, 93053 Regensburg, Germany; sebastian.einhauser@ukr.de (S.E.); ralf.wagner@ukr.de (R.W.); andre.gessner@ukr.de (A.G.); david.peterhoff@ukr.de (D.P.); 3 Institute for Clinical Microbiology and Hygiene, University Medical Center Regensburg, 93053 Regensburg, Germany; josef.koestler@ukr.de

**Keywords:** COVID-19, vaccination, BNT162b2, immunogenicity, reactogenicity, sex

## Abstract

It is not clear whether there is an association between adverse reactions and immune response after vaccination. Seven hundred and thirty-five vaccinees from our University Medical Center vaccination clinic provided information about sex, age and adverse reactions after first and second vaccination with BNT162b2. Adverse reactions were categorized into three groups: no or minor on the injection side, moderate (not further classified) and severe—defined as any symptom(s) resulting in sick leave. We chose 38 vaccinees with the most severe adverse reactions and compared their humoral and T-cell-mediated immune responses after second vaccination with those of 38 sex and age matched controls without or only minor injection-side related adverse reactions. Severe acute respiratory syndrome coronavirus 2 (SARS-CoV-2) anti-receptor binding domain (RBD) IgG titers were detectable in all participants (median 5528; range 958–26,285). Men with severe adverse reactions had 1.5-fold higher median SARS-CoV-2 RBD IgG titers compared to men without adverse reactions (median 7406 versus 4793; *p* < 0.001). Similarly; neutralization activity was significantly higher in men with severe adverse reactions (half maximal inhibitory concentrations (IC_50_) median 769 versus 485; *p* < 0.001). Reactogenicity did not influence humoral immune response in women nor T-cell-mediated immune response in any sex. To conclude; adverse reactions after vaccination with BNT162b2 do influence humoral immune response yet only in men and are not a prerequisite for a robust antibody response.

## 1. Introduction

It is a common belief that adverse reactions after vaccination are predictive signs of a good immune response; yet limited data is available. Vaccine reactogenicity characterizes the physical manifestation of the inflammatory response to a vaccine and can result in injection site and systemic symptoms. Some symptoms can be measured objectively, others are nonspecific and subjective. There are many factors that can influence reactogenicity; host-derived factors include age and gender, extrinsic factors dose number and injection technique [1]. Safety findings in the BNT162b2 phase 2/3 trial described reactogenicity as mild or moderate. Systemic reactogenicity was more common and severe after the second dose with events transient and resolving within a couple of days [2]. Real world data in users of the coronavirus disease (COVID) Symptom Study app in the United Kingdom found local side effects after BNT162b2 first and second vaccination with a frequency of 71.9% versus 68.5%, and systemic side effects with 13.5% versus 22.0%. Side effects were more prevalent in women and in vaccinees 55 years or younger [3]. The inflammatory response towards a vaccine normally leads into a measurable humoral and cell-mediated immune response. The most important immunological parameter in vaccine licensure is antibody levels [4]. For COVID-19 vaccines, binding and even more neutralizing antibodies are thought to be potential immune correlates of protection [5,6]. The significance of the cell-mediated immune responses against severe acute respiratory syndrome coronavirus 2 (SARS-CoV-2) is less understood. It is suggested that a balanced immune response of high titers of neutralizing antibodies and TH1-biased T-cells is probably optimal [7]. Whereas antibody responses appear to be short-lived, T cell memory is potentially more durable [8,9].

In the present study, we investigated whether there is an association between adverse reactions after vaccination with BNT162b2 and corresponding humoral and T-cell-mediated immune responses.

## 2. Materials and Methods

### 2.1. Recruitment of Participants

From 20 April–5 May, 1418 vaccinees—mostly health care workers and some university employees—received their second BNT162b2 vaccination in our University Medical Center vaccination clinic. After vaccination, they were informed about the study and asked whether they would be interested in participating. History of SARS-CoV-2 infection was an exclusion criterion. One thousand one hundred and ninety-seven subjects (84.4%) agreed to receive further information and were subsequently invited by E-mail to indicate their age, sex and adverse reactions after first and second vaccination. Adverse reactions should only be indicated according to the following classification:

(a) no adverse reactions or only minor on the injection side;
(b) moderate adverse reactions (not further classified);
(c) severe adverse reactions—defined as any symptom(s) resulting in sick leave or would have resulted in sick leave in case the vaccination was followed by day(s) off.

Seven hundred and forty-seven vaccinees responded. Twelve were excluded because of inconclusive information.

### 2.2. SARS-CoV-2 Binding Antibodies

Antibodies binding to the receptor-binding domain (RBD) of the spike protein of SARS-CoV-2 were quantified using an in-house enzyme-linked immunosorbent assay (ELISA) as described earlier [10]. Endpoint titers were determined by measuring eight two-fold serial serum-dilutions starting at 1:200. Exact titers were obtained from a curve fit using a sigmoidal four parameter logistic (4PL) regression.

Nucleocapsid protein-specific antibodies were quantified using a commercial line blot assay (Mikrogen, RecomLine Coronavirus IgG, Article No. 7372).

### 2.3. SARS-CoV-2 Neutralization Activity

Neutralization capacity of sera against SARS-CoV-2 was evaluated using the Vesicular Stomatitis Virus (VSV-Δ G*FLuc) pseudotyped with SARS-CoV-2-Spike-ΔER, which was previously proven to correlate well with SARS-CoV-2 neutralization [11].

Limited dilution and fluorescence microscopy were used to determine pseudoviral titers. For all samples, luciferase activity was determined 20 h post infection of HEK293T-ACE2+-cells, after neutralizing a fixed inoculum of 25,000 ffu for 1 h. Half maximal inhibitory concentration (IC_50_) values were calculated using the algorithm: ‘log (inhibitor) vs. normalized response’ in GraphPad Prism 8 software (GraphPad Software, San Diego, CA, USA). 

### 2.4. SARS-CoV-2 T-Cell Response

Venous blood was collected in lithium-heparin tubes and processed according to the instructions of the commercial T-SPOT.TB test (Oxford Immunotec, Milton Park, United Kingdom). Enzyme-linked immunosorbent spot (ELISpot) plates and chemistry were derived from the T-SPOT.TB test. Medium was used as negative control and phytohemagglutinin as positive control. A pool of overlapping S-protein derived peptides and two recombinant proteins, a prefusion-stabilized S-protein (StabS) and the S-protein’s RBD were used for stimulation. Recombinant proteins were produced and purified as described earlier [10]. ELISpot analysis additionally included peptide pools from SARS-CoV-2 membrane glycoprotein (M) and nucleocapsid phosphoprotein (N). Peptides from Miltenyi Biotec: SARS-CoV-2 Prot_S (Art.-No. 130-126-701), SARS-CoV-2 Prot_N (Art.-No. 130-126-699), SARS-CoV-2 Prot_M (Art.-No. 130-126-703).

Two hundred and fifty thousand peripheral blood mononuclear cells (PBMCs) were stimulated with the antigens. Antigen specific T cells released interferon-γ (IFN-γ) after contact with antigen presenting cells. The released IFN-γ bound to a precoated anti-IFN-γ antibody and, after adding a secondary labelled anti-IFN-γ antibody, was made visible as a spot by adding a detection reagent. The test was in house validated with five negative SARS-CoV-2 subjects and five SARS-CoV-2 positive patients. Positive IFNγ-ELISpot response was defined as at least eight spot-forming cells per 250,000 PBMCs.

### 2.5. Statistics

Analyses were conducted in Stata (16.0, StataCorp LLC, College Station, TX, USA). Owing to non-normal distribution Mann–Whitney U test was applied. An exact *p* value of 0.05 or less was considered to denote significance. Odds ratio (OR) was calculated by logistic regression. The 95% confidence interval (CI) was used to estimate the precision of the OR.

### 2.6. Ethical Issues

The study was conducted according to the guidelines of the Declaration of Helsinki and approved by the local Ethics Committee of the University of Regensburg (reference number: 21-2332-101). Informed consent was obtained from all subjects involved in the study.

## 3. Results

### 3.1. Selection of Participants

We intended to compare vaccinees with most severe adverse reactions after first and second vaccination with sex and age matched controls without adverse reactions or only minor injection side symptoms. Seven hundred and thirty five vaccinees were evaluated for participation in the laboratory analysis, 190 males (25.9%) and 545 females (74.1%). Table 1 indicates the adverse reactions after first and second vaccination with BNT162b2 in males and females.

More women suffered from adverse reactions after first (5.3% versus 1.6%) and second (38.3% versus 21.0%) vaccination. The OR for severe adverse reactions after first and/or second vaccination was 2.4 for women compared to men (95% CI 1.7–3.6).

We assigned participants into reactogenicity groups according to severity of adverse reactions after first and second vaccination (Table 2). We intended to include 90 participants in our study. To include most severely affected vaccinees, we invited 19 females from Group 1 and four females randomly selected from Group 2 that belonged to age groups underrepresented in Group 1. We invited two males from Group 1 and seven males from Group 2. Thirteen males were chosen from Group 4—randomly from age groups yet missing in the previous selection. For each participant, a same sex control (age ± 1 year) was randomly selected from Group 5.

Samples were taken from 7 June to 18 June. A questionnaire was handed out at enrolment. One participant who was selected from Group 5 reported headaches after vaccination and was excluded. Two participants were excluded because they reported a history of SARS-CoV-2 infection. Five participants did not show up for enrolment. As the enrolment process was ongoing, five of eight losses could be compensated by recruiting appropriate substitutes from the E-mail responders. After enrolment we decided to exclude three participants (and their controls) from the analysis because of immunosuppressive therapy. We excluded one participant (and control) because of a high value in the negative control of the ELISpot test. We finally included 76 vaccinees in our analysis.

### 3.2. Demography

Each reactogenicity group consisted of 20 males and 18 females. Median age was 43 years for vaccinees with severe adverse reactions and 42.5 years for vaccinees with no or minor injection side symptoms. Participants of the two groups were not different in respect to smoking, median body mass index, prevalence of any chronic disease and taking of antipyretic medication (paracetamol or ibuprofen) before vaccinations. Median time intervals between first and second vaccination and second vaccination and blood sample collection were equal. Characteristics of the two reactogenicity groups are shown in Table 3.

### 3.3. Humoral Immune Response

With a median titer of 5528 (range 958–26,285, IQR 2993), all vaccinees had SARS-CoV-2 RBD IgG titers within but mostly above the range of COVID-19 convalescent plasma donors at our University Medical Center (median 800, range 100–6400, IQR 1200, *n* = 41) [12].

**Figure 1:**
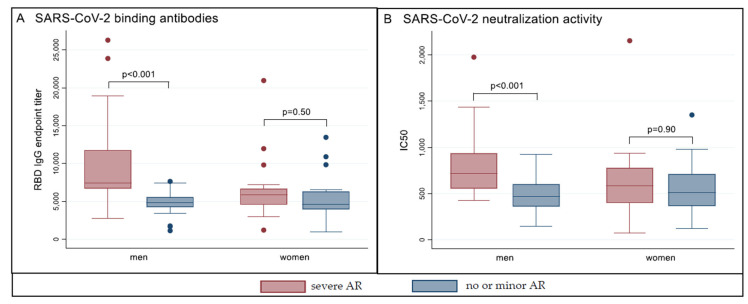
Antibody response and neutralization activity according to reactogenicity after second vaccination with BNT162b2. Shown are boxplots of RBD-specific SARS-CoV-2-IgG endpoint titers (Panel (**A**)) and half maximal inhibitory concentrations (IC_50_) in a pseudovirus neutralization assay (Panel (**B**), one outlier not included, leftmost bar IC_50_ 4231), separately in men and women. Dots indicate outliers. AR = adverse reaction(s). Severe AR = any adverse reaction(s) after first and/or second vaccination resulting in sick leave; no or minor AR = no or only minor injection side symptoms after first and second vaccination.

Participants with severe adverse reactions had 1.4-fold higher median SARS-CoV-2 RBD IgG titers compared to their controls (*p* < 0.001). Subgroup analysis showed that this difference was significant in men (median 7406 versus 4793; 1.5-fold; *p* < 0.001) but not in women (median 5892 versus 4628; *p* = 0.28) (Figure 1A). Similarly, median neutralization activity indicated in IC_50_ was 1.3-fold higher in vaccinees with severe adverse reactions (*p* = 0.005), while subgroup analysis confirmed improved separation in men (median 769 versus 485; 1.6-fold; *p* < 0.001) and no significance in women (median 583 versus 513; *p* = 0.63) (Figure 1B).

### 3.4. T-Cell Mediated Immune Response

**Figure 2:**
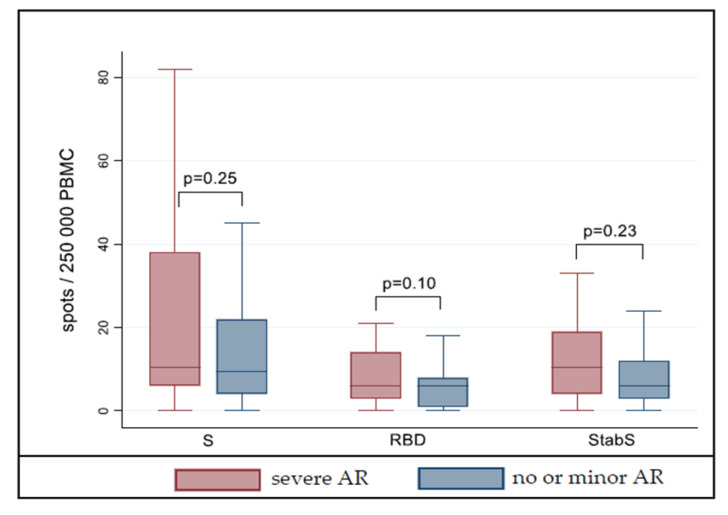
T-cell response according to reactogenicity after second vaccination with BNT162b2. Shown are enzyme-linked immunosorbent spot (ELISpot) results after stimulation of peripheral blood mononuclear cells (PBMCs) with S peptides (left), S protein receptor-binding domain (RBD, middle) and stabilized S protein (StabS, right) from SARS-CoV-2. Outliers not included. AR = adverse reaction(s). Severe AR = any adverse reaction(s) after first and/or second vaccination resulting in sick leave; no or minor AR = no or only minor injection side symptoms after first and second vaccination.

A T-cell-mediated immune response in ELISpot analysis was detectable in 30 vaccinees (79.0%) with severe adverse reactions and 25 vaccinees (65.8%) without or only minor injection side symptoms (*p* = 0.31). We did not see a significant difference in vaccinees of the two reactogenicity groups when surface glycoprotein S peptides (*p* = 0.25), RBD protein (*p* = 0.10) and stabilized S protein (*p* = 0.23) were compared (Figure 2). Subgroup analysis confirmed no difference in males and females (data not shown).

We had positive responses for SARS-CoV-2 membrane glycoprotein (M) in three participants of each reactogenicity group and one positive response for SARS-CoV-2 nucleocapsid phosphoprotein (N) in a participant without adverse reactions. A potential asymptomatic infection of those participants with SARS-CoV-2 reactive T-cells was ruled out by a negative result from a SARS-CoV-2 nucleocapsid protein-specific serology (data not shown).

## 4. Discussion

During the ongoing mass vaccination campaign, sick leave after vaccination was commonly described in the public. Hence for vaccinees who did not experience adverse reactions the question rose whether the vaccine really “worked” for them. Studies addressing a relationship between adverse reactions and immunogenicity are rare.

Burny et al., compared reactogenicity and inflammatory markers after HBsAg-adjuvanted vaccines and found significant associations between systemic symptoms and IL-6 and IFN signals that were previously shown to be associated with the adaptive response [13]. For whooping cough, the change from the whole cell vaccine with pronounced inflammatory reactogenicity to the better tolerated acellular vaccine is supposed to be responsible for the re-occurrence of whooping cough in the vaccinated population due to reduced immunogenicity of the acellular vaccine [14].

Previous studies investigating immunogenicity and reactogenicity of COVID-19 vaccines found inconsistent results. Naaber et al., described a significant association of a total score of adverse effects with S-RBD IgG levels [15]. Other studies could not find an association [16,17,18]. None of the studies chose participants according to adverse reactions but compared adverse reactions and antibody levels in a population at hand. For our study, we selected the most severely affected vaccinees from a recruitment population of 735 and compared them with controls who indicated no or only minor injection side symptoms after both vaccinations. When planning our study, we were thinking of a severity score for adverse reactions but found it rather difficult to value symptoms. Signs of vaccine reactogenicity are often subjective and therefore difficult to evaluate [1]. We finally opted for sick leave as a surrogate for severe adverse reactions of any kind. This may be an even more objective surrogate in countries where there is no sick pay.

The association between reactogenicity and antibody levels in men was striking. Yet sex is known to play an important role in immune response to self and foreign antigens. Females are more prone to autoimmune diseases and vaccine-induced adverse reactions. Their antibody titers are higher after many vaccinations. On the contrary, men are more susceptible to non-reproductive malignant cancers. Finally, infectious diseases vary among both sexes. These differences can be attributed to the influence of environmental factors, sex chromosome genes and sex hormones on immune responses [19]. In our recruitment population of 735 vaccinees, sick leave after vaccination with BNT162b2 occurred more often in women than in men. Actually, females in the adverse reaction group were “sicker” than corresponding males because the majority (13/18) reported sick leave after first and second vaccination whereas most males reported sick leave only after second vaccination (14/20). Yet our pool of males was considerably smaller (190 males versus 545 females) [20].

We could not detect a difference in SARS-CoV-2 RBD IgG, neutralization activity and T-cell response when comparing males and females independently from adverse reactions (data not shown). Findings in other studies with BNT162b2 are inconsistent [21,22,23]. Likewise, it remains inconclusive how sex correlates with SARS-CoV-2 antibody response in COVID-19 patients as studies in convalescent plasma donors describe different outcomes [24,25,26]. We assume that selection bias plays an important role when comparing antibody levels—both in vaccinees and convalescent plasma donors—and may lead to opposite findings.

BNT162b2 elicited a robust cell-mediated immune response in a phase 1/2 trial [27]. We had a higher proportion of positive ELISpot results in the adverse reaction group but could not show significance. Possibly our sample size was too small to detect a slight difference.

Whether males with severe adverse reactions after BNT162b2 will maintain a superior antibody response is subject to further investigation. Because of the recruitment of participants from the working population, information is missing if the observed phenomenon also applies to other age groups. We had a large range of laboratory outcomes that may have influenced our analysis. We finally do not know whether our results are applicable to SARS-CoV-2 vaccines other than BNT162b2. A larger cohort is necessary to confirm our preliminary results.

## 5. Conclusions

This is, to our knowledge, the first study that clearly showed a sex-dependent relationship between adverse reactions and vaccine-induced antibody response with severely affected men having higher SARS-CoV-2 RBD IgG titers and neutralization activity compared to men with no or only minor injection side symptoms. In contrast, T-cell response was not influenced by reactogenicity. The most important message from our study is, however, that all vaccinees developed a reasonable antibody response independently from reactogenicity.

## Figures and Tables

**Table 1:** Adverse reactions after BNT162b2 vaccination in the recruitment population. IQR = interquartile range.

Characteristic	All *n* = 735	Male *n* = 190	Female *n* = 545
median age in years (range, IQR)	38 (16–66, 25)	42 (16–66, 26)	36 (16–66, 23)
adverse reactions after 1st vaccination (%)			
no/minor at injection side	551 (75.0)	155 (81.6)	396 (72.7)
moderate	152 (20.7)	32 (16.8)	120 (22.0)
severe	32 (4.3)	3 (1.6)	29 (5.3)
adverse reactions after 2nd vaccination (%)			
no/minor at injection side	270 (36.7)	87 (45.8)	183 (33.6)
moderate	216 (29.4)	63 (33.2)	153 (28.1)
severe	249 (33.9)	40 (21.0)	209 (38.3)

**Table 2:** Reactogenicity groups relevant for the selection process separated by sex.

	Group 1		Group 2		Group 3		Group 4		Group 5	
Adverse Reactions after 1st/2nd Vaccination	Severe Severe		Moderate Severe		Severe Moderate		No or Minor Severe		No or Minor No or Minor	
sex	male	female	male	female	male	female	male	female	male	female
all responders (*n* = 735)	2	19	7	59	0	6	31	131	76	149
participants included in analysis (*n* = 76)	1	13	5	5	-	-	14	-	20	18

**Table 3:** Characteristics of vaccinees according to reactogenicity group. AR = adverse reaction(s); IQR = interquartile range; BMI = body mass index.

Characteristic	Severe AR (*n* = 38)	No or minor AR (*n* = 38)	*p*-Value
male sex—no. (%)	20 (52.6)	20 (52.6)	1.00
median age in years (range, IQR)	43 (23–64, 26)	42.5 (23–63, 25)	0.97
adverse reactions after 1st vaccination			
no/minor	15 (39.5)	38 (100)	<0.001
moderate	7 (18.4)	0	0.01
severe	16 (42.1)	0	<0.001
adverse reactions after 2nd vaccination			
no/minor	0	38 (100)	<0.001
moderate	0	0	1.00
severe	38 (100)	0	<0.001
smoking	4 (10.5)	1 (2.6)	0.36
median BMI (range, IQR)	23.7 (18.4–32.4, 4.7)	24.8 (17.6–35.9, 6.0)	0.40
any chronic disease	9 (23.7)	10 (26.3)	1.00
immunosuppression	0	0	1.00
antipyretic medication before 1st vaccination	1 (2.6)	2 (5.3)	1.00
antipyretic medication before 2nd vaccination	4 (10.5)	1 (2.6)	0.36
median time interval between 1st and 2nd vaccination—days (range, IQR)	33 (21–38, 12)	33 (21–38, 12)	0.84
median time interval between 2nd vaccination and blood sample collection—days (range, IQR)	49 (35–58, 5)	49 (35–58, 7)	0.97

## Data Availability

The data sets used and/or analyzed during the present study are available from the first author on reasonable request.
